# To treat or not to treat: Experiences and considerations of veterinarians in management of cats with diabetes mellitus

**DOI:** 10.1371/journal.pone.0341762

**Published:** 2026-02-05

**Authors:** Ninni Rothlin-Zachrisson, Bodil Ström Holst, Malin Öhlund, Janeth Leksell, Helena Röcklinsberg

**Affiliations:** 1 Department of Clinical Sciences, Swedish University of Agricultural Sciences, Uppsala, Sweden; 2 Swedish Medical Products Agency, Uppsala, Sweden; 3 Department of Medical Sciences, Uppsala University, Uppsala, Sweden; 4 Department of Applied Animal Science and Welfare, Swedish University of Agricultural Sciences, Uppsala, Sweden; Universidade de Trás-os-Montes e Alto Douro: Universidade de Tras-os-Montes e Alto Douro, PORTUGAL

## Abstract

Veterinarians’ daily work involves navigating ethical, medical, and emotional considerations while balancing diverse perceptions of responsible care within the triadic relationship between veterinarian, animal, and owner. These challenges are particularly evident in the management of chronic diseases. Diabetes mellitus serves as an example in our study, where we explore Swedish veterinarians’ experiences of managing it in cats. Individual in-depth interviews were conducted with ten veterinarians, and the data were systematically analysed using reflexive thematic analysis. One overarching theme, the perceived prioritisation of the cat’s wellbeing, was identified as central to how veterinarians approached the clinical situation. Three themes are addressed: the role of the owner in disease management, the negotiation between medical ideals and practical realities, and the perception of euthanasia as being in the best interest of the cat. Taken together, the three themes explore how veterinarians continually negotiate what it means to act responsibly within the constraints of real-life practice. This ongoing negotiation between ideals, pragmatism, and compassion underscores the ethical depth and emotional labour embedded in everyday veterinary work. The results contribute in-depth insights into how clinical reasoning and decision-making is shaped by both moral positions and pragmatic adaptations, and the importance of veterinary-owner communication in finding the optimal management plan in each individual case. Awareness of the challenges associated with diabetes care in cats may facilitate ethical reflexivity in veterinary practice, supporting decisions that impact both animal welfare and veterinarians’ professional wellbeing.

## Introduction

Companion animals play a significant role in human lives [1–4]. As the provision of care for animal patients’ health and welfare is a core principle of the veterinary profession [5–7], veterinarians are central to sustaining and supporting human–animal interactions. However, beyond the frameworks established by legislation, perceptions of what constitutes “responsible care” vary considerably. Individuals differ in how they interpret their obligations towards animals, including what is believed to be in the animal’s best interest, how far these responsibilities extend, and how they are best fulfilled [8–10]. What one person considers to be an appropriate and justified course of action may not align with another’s view [5]. For veterinarians, this diversity of beliefs and expectations gives rise to a wide range of morally challenging situations in daily practice [11–13], which requires balancing the animal’s needs, the owner’s preferences and capacities, and the veterinarian’s own professional and ethical commitments. This balance is widely regarded as one of the most multidimensional moral challenge in veterinary medicine [14,15]. In light of this, it is perhaps unsurprising that moral stress, arising from an inability to act in accordance with one’s own moral beliefs [16], and compassion fatigue are increasingly discussed as important factors in veterinary mental health [13,17–20]. Gaining insight into how veterinarians perceive moral challenges in their daily practice, and the processes through which they make sense of associated experiences, is important for a comprehensive understanding of the realities of veterinary practice.

In modern veterinary medicine, this complexity is heightened by a growing acceptance of advanced medical options for animals, perhaps reflecting the shift in their status from property to family members [2,21,22]. This is particularly evident in the management of diabetes mellitus (DM) in cats, a common chronic endocrine disorder [23–26] that requires medical intervention and careful long-term management. With effective treatment, typically involving regular hypoglycaemic therapy (e.g., insulin injections), dietary changes, lifestyle adaptations, and home monitoring, many cats can achieve satisfactory glycaemic control, maintain a good quality of life, and live for years beyond their diagnosis [27–30]. In some cases, cats who respond well to treatment may achieve diabetic remission, eliminating the need for ongoing hypoglycaemic treatment.

Treatment goals for DM include both the potential for remission and the establishment of a sustainable management plan that is acceptable to the cat and feasible for the owner to maintain over time [31–33]. Successful management of DM depends on the owner’s ability and willingness and entail a high degree of responsibility on the owner at home. Euthanasia remains a possibility, especially around the time of DM diagnosis [28,29,34,35]. Medical prognosis and quality of life are important factors in the decision as to whether to euthanise chronically ill companion animals [28,36,37]. In addition, contextual factors related to both veterinarians and owners, including owners’ perceived ability to cope with the demands of treatment, including emotional, financial, and practical challenges, is influential in clinical decision-making [38–41]. Thus, the successful management of DM in cats extends beyond clinical protocols, and veterinary care in this context unfolds within the triadic relationship between the veterinarian, the cat, and the owner [42,43].

Despite the recognition of the veterinarian’s role [14,41,44], there remains a lack of in-depth research exploring how veterinarians perceive and respond to these factors in practice [45–48]. DM management necessitates continuous owner involvement and veterinary oversight, and places veterinarians in a key position to perceive and respond to the interactions between treatment requirements, owner circumstances, and animal health and welfare throughout the course of the disease. This study aimed to deepen the understanding of the considerations, decision-making processes, and lived experiences of Swedish veterinarians caring for cats diagnosed with DM. Particular attention was given to veterinarians’ professional responsibilities, interactions with owners, and the challenges encountered in daily practice.

## Methodology

The study was based on individual in-depth interviews with ten veterinarians, all with experience in diagnosing and managing cats with diabetes mellitus. Before initiating the study, an ethical review was conducted and approved by the Swedish Ethical Review Authority (Dnr 2021−00939). See S1 Appendix for a detailed description of methodological considerations, reflexivity and coding structure.

### Recruitment process and participants

Participant recruitment involved a combination of convenience sampling and purposive sampling [49]. Veterinarians were recruited by posting a call for participants in two Swedish Facebook groups with over 2900 and 450 members respectively, including veterinarians employed in different fields of veterinary medicine. Veterinarians expressing interest in participating were asked to provide information regarding their clinical experience (years in clinical practice) and workplace (e.g., small animal practice, mixed animal practice, referral clinic), and where they received their veterinary education. Inclusion criteria were: being a veterinarian and having experience managing cats diagnosed with DM and their owners. Participation was voluntary, without compensation, and each participant received written information about the study, data management, and an informed consent form. The recruitment process began on April 30, 2021, and concluded on May 16, 2021. Information about the research project, including the primary author, was provided.

The number of participants was guided by the concept of information power [50], and Braun and Clarke’s reflections on data collection within reflexive thematic analysis [51], and were assessed stepwise and continuously during the course of data collection. First, five interviews were conducted, and field noted were used to assess the data for differing experiences and data quality in relation to the research questions. Based on this and in search to diversify perspectives, two veterinarians were directly approached by the primary author in the Facebook groups, and, later, two additional veterinarians were recruited through personal contacts. After nine interviews, the data was assessed as rich enough to proceed with analysis. To avoid prematurely closing data collection, one additional interview was conducted. To reduce the risk of earlier data influencing later interviews, transcription and coding were postponed until all interviews were completed.

A total of ten veterinarians were recruited, the majority of whom were female (9/10). The participants represented different geographic regions of Sweden, and, at the time of the interviews, most worked exclusively in small animal practice. Some had experience of mixed animal practice. Their professional backgrounds varied, encompassing different types and sizes of clinics, including both primary care and referral practices. All but one of the participants had had their degree of veterinary medicine issued in Sweden. For an overview of veterinarians´ characteristics, see Table 1.

**Table 1:** An overview of participating veterinarians’ characteristics.

Participant*	Age** (years)	Gender	Years since graduation
Ann-Marie	65	Female	40
Carolina	35	Female	5
Emma	30	Female	5
Emelie	40	Female	10
Helen	40	Female	10
Karin	50	Female	10
Karl	50	Male	20
Katarina	60	Female	30
Kerstin	40	Female	5
Lotta	35	Female	10

*Pseudonym.

**Approximated to nearest five.

### Data collection

In-depth individual interviews were selected, allowing for flexibility in questioning and follow-up and enabling participants to elaborate on specific situations or lines of reasoning [49,52]. Interviews were conducted in Swedish via the video and audio software Zoom. Verbal consent was obtained prior to recording; one interview was conducted without video recording at the participant’s request. All interviews were carried out by the first author, with no third parties present.

The interviews were semi-structured and guided by a pre-developed interview protocol (see S2 Appendix). Most questions were open-ended to minimise interviewer influence and encourage rich, participant-driven narratives. The guide was piloted with two veterinarians and revised for clarity and content prior to main data collection. While all topics were covered in each interview, the sequence of questions was adapted to suit the natural flow of discussion. Member checking was applied continuously during the interviews, allowing participants to correct and confirm data interpretation [53]. Interviews lasted approximately 50–60 minutes. Fieldnotes were written after each session to capture immediate impressions.

The video-recorded interviews were transcribed manually and verbatim by the first author using oTranscribe, following a consistent protocol for non-verbal cues (e.g., laughs, nods) to enhance transcription accuracy [54]. The average transcript length was 6578 words (range: 4113–9133). Transcription was deferred until all interviews were completed. The transcriptions were not returned to participants for correction. However, participants were invited to contact the researcher post-interview with any clarifications, concerns, or requests to withdraw. Relevant excerpts and quotations from the transcripts were later translated into English by the first author. Pseudonyms were assigned to all participants to ensure confidentiality during analysis and reporting.

### Analytic process

A six-phase reflexive thematic analysis was used to identify and develop patterns of experience and meaning across the dataset [55,56]. The study was grounded in a constructivist paradigm, recognising that knowledge and meaning is shaped by interactions, language, and context. This encompasses individuals’ prior experiences and upbringing, as well as the specific circumstances in which a situation is interpreted [57], understanding reality as individually constructed, rather than objectively fixed. An experiential orientation guided the research, with a focus on participants’ lived experiences and personal meanings. A reflexive methodology supported this approach [49,56], with the research team’s subjectivities used to enrich interpretation and maintain sensitivity to participant perspectives.

The analytic process involved both digital tools (Delve) and hard-copy materials to accommodate different phases of interpretation. Each analytic step was carefully documented. Initial familiarisation involved detailed reading and annotation of the transcripts. Coding of topic of interest involved both semantic and latent elements, beginning with a primarily descriptive approach to capture participants’ explicit expressions, and including more latent-level codes in segments where deeper insight was required. Codes were developed iteratively and were refined across multiple rounds of analysis. Two transcripts were independently coded by the first author and a co-author to enhance reflexivity and stimulate discussion around alternative interpretations. Once coding was complete, themes were developed by clustering related codes that reflected recurrent patterns across the dataset. The themes were refined through comparison with the data and checked for consistency with the corresponding quotes to ensure accurate representation of participants’ perspectives. Writing was an integral part of the analysis. In the reflexive process, including considerations of researcher positionality and its potential influence on data engagement [56], ongoing reflection and discussions between co-authors helped to broaden researcher perspectives, facilitate inclusivity in perspectives and reduce preconceptions.

## Analysis and discussion

### Thematic structure

We created one overarching theme; *at the core of veterinary responsibilities*, that relates to veterinary prioritisation of the cat’s well-being and tied together three themes constructed from the data. The three themes form a coherent moral landscape, where veterinarians continuously negotiate the boundaries between what ought to be done, what can be done, and what feels bearable to do. In this negotiation, the cat’s welfare, the owner’s situation, and the veterinarian’s own emotions are in constant dialogue. The first theme*, the owner is the key,* explored the narrative of how veterinarians approach the role of the owner in decision-making, recognising that the potential for treatment success relies on the owner’s willingness and ability to engage in the cat’s care. The second theme*, a chance to live,* discusses how veterinarians balance medical ideals with practical realities, engaging with the owner in hope of achieving a successful treatment outcome. The third theme*, better dead than suffering,* concerns euthanasia as ensuring no unnecessary suffering is caused, and how veterinarians navigate the associated professional and emotional challenges. For an overview of the thematic structure, see Table 2.

**Table 2:** Thematic structure and connections in veterinarians’ experiences of treating diabetes mellitus in cats.

Analytic structure:	Description:	Examples of sub-themes and key data extracts:
**Overarching theme**		
At the core of veterinary responsibilities	Veterinarians’ perceived responsibilities towards cats and owners.	A patient in its own right: *“My focus is always on the cat.”* Cat and owner wellbeing: “*The team of both needs to be prioritised.”*
**Themes**		
The owner is the key	How veterinarians approach the role of the owner in decision-making.	The owner plays a central role: *“Our responsibility towards the animal won’t be fulfilled if we don’t satisfy the owner.”* Communication matters: “*It´s important to de-dramatise it*.”
A chance to live	Engaging with owners and adapting a flexible approach to give the cat a chance at treatment.	Introduce treatment stepwise: *“You can become stricter later.”* Deviations from the treatment regimen: *“I revise my plan based on what the owner wants.”*
Better dead than suffering	Euthanasia as the best interest of the cat while navigating associated professional and emotional challenges.	Quality before quantity: *“This is from the cat´s perspective.”* Coping mechanisms: *“A protective barrier for your psyche.”*

### Overarching theme: At the core of veterinary responsibilities

Participants consistently framed the cat as the primary centre of veterinary care, recognizing their professional role as advocates for the cat. Emelie explained how *“it’s extremely important, their [the cats’] quality of life. […] We are responsible for ensuring that they feel good”*. By explicitly framing responsibilities around the cat’s well-being, clinical actions can be better aligned with what is perceived to be in the cat’s best interest, suggested to include avoiding harm, upholding health, and promoting both the quantity and quality of life of the cat [5,58]. This focus may also be what owners expect of their attending veterinarian [59], and supports the normative claim of prioritization the animal´s interests in veterinary medicine [44,58,60]. Additionally, participants described how they to various degrees included the owner while navigating their practice, positioning their responsibilities as dual. Carolina stated: “*If you have a healthy cat, you have a healthy owner; I often think it goes together.”* For Ann-Marie “*the first and most important thing is the cat and the owner together.”* Recognising the owner’s wellbeing as inseparable from that of the cat suggests how a holistic approach to care could benefit both parties, an interaction that has been recognised as particularly important to consider when supporting owners of chronically ill animals [61]. However, advocating for the cat’s best interests while simultaneously recognising the owner carries an inherent conflict [62], as it may require negotiation between prioritising the cat’s welfare and acknowledging the owner’s needs. Participants’ awareness of their responsibilities laid the foundation for further reasoning regarding DM management and interventions.

### Theme 1: The owner is the key

The first theme explored how veterinarians considered DM management in relation to the owner’s willingness and ability to engage in care. While regarding themselves as stewards of the cat’s welfare, there was a general acknowledgement that this obligation could only be realised through working with the owner. Karl speaks of the importance of nurturing a positive relationship with the owner:

*“The well-being of animals and our responsibility towards them won’t be fulfilled if we don’t satisfy the owners. It’s impossible to ensure that the animals are well if we don’t engage with the owners. […] The owners need to trust us and be happy with what we do; otherwise, we won’t be able to provide the care the animals truly need.” (Karl)*

This rather pragmatic view reflects how owners hold final authority over treatment and euthanasia decisions upon DM diagnosis, and how the owners’ experience of veterinary care is central to both the veterinarian–client relationship [63] and decision-making [64]. This approach aligns with a broader definition of successful disease outcomes that includes care that corresponds with the individual owners goals and expectations [59]. Hence, establishing a connection with the owner is not a distraction from the focus on the cat, but is rather a necessary step to ensure that the cat receives proper care. It has been emphasized how owners must trust the veterinarian’s guidance and also feel confident and be supported in their own ability, otherwise the likelihood of achieving successful treatment outcomes diminishes [65,66]. One strategy for enabling such collaboration was careful attention to the owner’s perspective, and Emelie explains how “*you need to show the owner that you see them, that you understand them in this situation”*. A sensitivity towards their experiences recognises how owners’ confidence and emotional well-being may directly shape the cat’s outcome. By acknowledging the weight of decision-making and validating feelings of overwhelm or worry, veterinarians may create a space where owners feel heard and respected [67], aligning with an empathy-driven owner support [68]. This sensitivity towards the emotional state of the owner was also articulated through awareness of a potential disconnect between a professional familiarity with treatment and the owner’s fear or discomfort: “*It’s easy for me to say, as someone who is used to injecting animals, that this is really simple and nothing to worry about.”* (Emma). Some participants stressed “de-dramatising” DM by encouragement and positive framing of previous treatment outcomes and described sharing their experiences of DM as a manageable disease in order to reduce owner anxiety and strengthen confidence. These strategies represent different ways of engaging with owners, and share the goal of fostering trust and building a partnership, an important condition for treatment adherence and, ultimately, for preserving the cat’s quality of life [65]. Others emphasised respect for owner autonomy and moral agency:

*“My strategy is generally to provide a lot of information—about what the disease is, what it entails, what lifestyle changes it means for the owners and the cat, and what needs to be done in terms of treatment; whether that’s taking blood samples, giving insulin, changing the cat’s diet, and so on. So that they have all the information and can make an informed decision about whether this is something they believe they can and want to treat.” (Lotta)*

This account suggests a strategy that operates on the presumption that owners will act not only in their own best interest but also in what they perceive to be the best interest of their cat, even if their decisions do not necessarily align with the veterinarian’s moral perspective. Clear explanations of recommendations may influence how owners perceive the value and quality of care, while a lack of understanding about the necessity of a recommendation is a key reason for noncompliance [69]. This may also represent an example of how limiting the provided information could encourage owners to search for potentially non-reliable or misleading information elsewhere, e.g., online, which could have a negative impact on the veterinarian–owner relationship and, correspondingly, the cat’s quality of life [70]. Participants’ accounts of communication with owners at the time of a DM diagnosis demonstrates an awareness of recognised owner concerns associated with DM treatment [28,38,71,72]. Across strategies, the underlying reasoning remained consistent: access to the cat’s wellbeing is mediated through the owner. This suggests that veterinary communication style both reflects the dynamic of the relationship with the owner, e.g., a more paternalistic or a shared approach [73], and is indicative of how they approach the owner as means to reach the outcomes they assess as most beneficial for guarding the cat’s quality of life.

### Theme 2: A chance to live

The second theme conceptualises veterinarians’ accounts of engaging closely with owners and modifying management protocols to avoid euthanasia and make treatment feasible for both owner and cat. A prerequisite for allowing modifications was assessing the cat as having a reasonable chance of responding well to treatment: “*It might be overweight, eating dry food, and on corticosteroids, and everything points to being able to get this cat into remission.*” (Kerstin). Commonly, the reasoning was framed in terms of offering the cat a chance of treatment and, ultimately, a chance to live, even when owner’s circumstances hindered implementing the desired treatment regimen. A key to this approach can be found in how the owner’s decision upon the cat’s DM diagnosis as not merely a binary choice between an optimal treatment regimen (commonly described to include “an immediate switch of diet” and “insulin injections”) and euthanasia. Alternative management strategies, although potentially suboptimal in terms of maximising clinical outcomes, may still offer a reasonable and meaningful path forward for both the cat and the owner [74]. Participants described how their own or others’ experiences of successful clinical outcomes served as motivation: if some cats have thrived under treatment, withholding that opportunity from others may feel unjustified. They reported employing incremental strategies, such as initiating dietary changes prior to insulin therapy or simplifying DM monitoring, to facilitate adherence and motivate owners to commence treatment:

*“I’m not very strict, so to speak. Rather, I feel that if you can just get things started and everything flows smoothly, then you can become stricter later if needed, once they [the owners] are on track. […] Pricking for blood every day… If you say that, you might scare some owners away.”* (Susanne)

By implementing tailored strategies to address challenges, participants aimed to enhance owner compliance and engagement while still fulfilling their perceived obligations to the cat. Adaptations in the desired management plan was implemented both proactively, without owners explicitly requesting adjustments, and in direct response to owners expressed wishes and needs. Alleviating some of the concerns about the demands of treatment can increase the likelihood of sustained adherence [75] and reduce the risk of euthanasia driven by uncertainty or apprehension of caregiver burden [39,76]. Based on prior experiences of how owners react and perceive DM management, proactive adjustments may increase the likelihood that owners would initiate treatment but could risk blurring the line between supporting and steering the owner. That risks limiting the owners’ role in decision-making and challenges their autonomy [73]. In addition to adaptations serving as motivators to commit to care, there was generally a high level of acceptance for various reasons to accommodate the owner’s needs, including owners’ lifestyle (e.g., irregular working hours or owning multiple cats), capacity (e.g., fear of needles), and competing responsibilities (e.g., caring for children or sick family members). However, one constraint was often viewed as particularly challenging:

*“It’s especially difficult when it’s the financial aspect that falls short. […] You try to make something out of nothing, by finding something that might not be gold standard, or even silver standard, but at least a bronze standard.”* (Emma)

In accounts describing this, frustration appeared to arise not towards the owner, but from the tension created by financial constraints setting absolute limits to what could be offered, imposing boundaries that could not be negotiated or modified in the same way as, e.g., lifestyle-related factors. Highlighting a medically desired option (“gold standard”) suggests that the revised outcome may still be deemed acceptable, and compromising can be seen as an attempt to balance moral imperatives towards both the cat and the owner. Described modifications included reducing veterinary visits, accepting less stringent disease control to lower the risk of insulin overdose, or relying solely on a low-carbohydrate, high-protein diet despite the potential added benefit of insulin. As reported elsewhere [20,41], financial obstacles were perceived as very stressful, and left veterinarians feeling insufficient in their obligations towards the cat. However, in contrast to results from a survey in Canadian and UK veterinarians [77], veterinarians emphasised that they did not adjust medical advice based on presumptions of an owner’s finances, a difference that may be explained by the higher proportion of insured cats in Sweden (approximately 60% in 2023) [78]. Possibly, it could also reflect a response to owners’ expectations that the veterinarian should prioritise the animal’s well-being over cost considerations [79].

### Theme 3: Better dead than suffering

The third theme focuses on the veterinarian’s reasoning surrounding euthanasia. Overlapping but distinct framings of euthanasia were constructed, each reflecting how veterinarians negotiated their responsibilities towards the cat, the owner, and themselves. Reflections included experiences from both from when DM was first diagnosed and from later stages of disease management. Both euthanasia due to the cat’s condition and from the circumstances related to the owner’s willingness and ability to ensure adequate medical care were discussed. Many accounts gave the sense that euthanasia was not perceived as a failure, but rather a pragmatic decision:

*“Above all, perhaps, when it comes to recommending euthanasia...When I think that this is not okay, we can’t do this... That is from the cat’s perspective.” (*Lotta)

“*Happiness and suffering are the measures that matter, and whether to live or die I don’t really see as a major factor in what I do — avoiding suffering is really what we’re doing*.” (Karl)

By positioning euthanasia as a decision made considering the cat, the focus shifts from external circumstances to the animal’s lived experience, suggesting euthanasia as a morally acceptable act. In contrast to previous definitions of ethically indicated euthanasia [9], veterinarians in our study seemed to extend the reasoning beyond previously described non-acceptable motivators, such as convenience or cases involving sick animals manageable with standard care [80]. While DM may not fully fit this category, a focus directed toward the cat’s overall interests, well-being, and potential for suffering, reflects how euthanasia may be regarded as morally acceptable when its primary purpose is to protect the animal’s welfare and prevent suffering, regardless of the underlying cause. Although both the Swedish Animal welfare act (2018:1192) and animal welfare research [81] include the animal’s positive mental state as essential for their welfare, the minimisation of negative experiences (e.g., suffering) remains a primary concern. In this context, euthanasia can be seen as the epitome of veterinary responsibility [82], providing a peaceful passing, or a “good death” [83]. Further, in some accounts, euthanasia was presented as a response to insurmountable owner limitations, such as severe illness, where even the most basic treatment could not be provided. Ann-Marie described how “*we talked about injections, and she showed me her hands. ‘Do you think I can handle a syringe?’“.* Others spoke of an uncompromising stance, in which full owner commitment to tight insulin protocols, blood glucose measurements, and regular visits was considered a prerequisite for initiating treatment:

“*If you feel that you don’t have the ability, for whatever reason, […] then we shouldn’t start. There’s no point in starting insulin if you’re not dedicated and willing to work with this, then it’s better to just leave it.*” (Karin).

This “all-or-nothing” approach to DM management suggests a strong advocacy for cat quality of life, with the cat´s interests safeguarded by protecting it from potential upcoming suffering. This resembles euthanasia decisions informed by veterinarians’ prior negative experiences with the quality of care provided by some owners [84]. The non-negotiable position contrasts with a survey of small animal veterinarians in North America [20], where the animal’s legal status as property made veterinarians regard providing potentially non-beneficial care as a duty, suggesting different cultural approaches to how animals are viewed in this context. In addition, the position may also include consideration for the owner, in how initiating a burdensome treatment with a deemed low likelihood of success could be seen as unjustifiable, however at the cost of owner autonomy [14]. While a veterinarian may refuse euthanasia if the cat is healthy [85], it was clear that participants shared a professional and moral obligation to perform it when the cat was suffering or treatment was no longer a viable option. Although commonly approaching euthanasia with a practical perspective, there was a noticeable sense of regret when owners, for various reasons, chose not to initiate treatment:

*“I can understand…It’s not for everyone, and they’re taking responsibility as owners by not letting it [the cat] go untreated or simply ignoring it, but it still hurts the heart. […] This is a treatable disease in many ways. It’s a shame that this cat didn’t get that chance.” (*Helen)

In this context, euthanasia can be framed as a ‘second-best’ option. The cat might have lived well under different circumstances, but under non-ideal conditions, euthanasia is reframed and neutralised as the most responsible course of action, resembling the concept of contextually-justified euthanasia [86]. Accounts like these acknowledge the structural limits of veterinary care, including owner capacity, financial constraints, and the broader social context [60]. Navigating these clinical situations, where empathy for the owner may coexists with a sense of moral discomfort, can be a significant source of stress, a concern that has increasingly been recognised as an issue within the veterinary profession [13,17,20]. Whether consciously or not, participants described a subtle detachment from owners and their decisions: *“You can’t get personally involved in every case. […] You need some kind of protective barrier for your own psyche”* (Kerstin). This stance suggests a form of professional distancing as means of coping with challenges in clinical practise, such as financial limitations or emotional difficulties faced by owners.

However, the cumulative exposure to conflicting perspectives and constrained situations can reinforce a tendency to prioritise detachment [20]. Keeping a professional distance from the clinical situation to encourage rational decision-making and emotional regulation has been described as a tool for long-term work–life balance [87], but may also become problematic, as reliance on emotional detachment may result in reduced empathy-driven care [88]. When veterinarians prioritise distance, they may overlook important contextual factors affecting owners, factors that play a role in treatment decisions. Although this protective mechanism might alleviate the experience of ethical conflicts, it risks undermining the veterinarian’s capacity to advocate effectively for the animal’s best interests or reduce openness to the role of emotions in the decision-making.

### Summary and clinical importance

This is the first study to qualitatively investigate how veterinarians approach DM management in cats and how they deal with related challenges in clinical practice. The inclusion of veterinary in-depth perspectives provided new insights into clinical management of DM and addresses a limitation with previous research, where attention has primarily been given to the owner´s perspective. Together, the three themes capture how veterinarians manage the tension between professional ideals, practical realities, and emotional responsibility in the management of diabetes mellitus in cats. This dynamic interplay characterises the moral complexity of everyday veterinary practice. We identified how disease management was shaped by veterinary responsibilities primarily to the cat, but also to the owner, who was also considered essential for ensuring the cat’s quality of life. Clinical decisions were influenced by owners’ circumstances, and veterinarians’ responsibilities were manifested either by offering different protocols for enabling treatment, or by protecting the cat from non-beneficial care and recommending euthanasia. Veterinary clinicians may benefit from the insights on how communication between veterinarian and owner is key for finding the optimal management plan in each individual case. Awareness of the potential ethical challenges for veterinarians facilitate discussions and ethical reflexivity which may render veterinarians better equipped for handling these and similar challenges in animal health care.

### Limitations and suggestions for future research

Efforts were made to ensure diversity among participating veterinarians, including variation in gender, age, geographical location, place of work, and clinical experience with diabetes mellitus in cats. Despite these efforts, it proved challenging to recruit a more demographically diverse group; for instance, the majority of participants were female. This may reflect the general demographic composition of the veterinary profession, with a greater proportion of veterinarians being women both in Sweden and internationally [89,90]. A more diversified sample might have resulted in differing perspectives on the issues discussed [13,77,91]. Additionally, participants may reflect a particularly motivated or reflective group within the veterinary profession in Sweden, and as such, may have shaped the nature and depth of the generated insights.

The exclusive inclusion of Swedish veterinarians limits the transferability of the findings. In Sweden, veterinary costs are paid by the owner. Beyond health insurance, few other options of funding exist (e.g., charity or trust funds), most likely because of the high proportion of insured companion animals. Owners’ financial limitations in relation to treatment decisions and veterinary health have been studied in veterinarians practicing in the UK, Denmark, and North America [20,41,92]. The results from this study suggest further investigation of owner financial limitations in the context of chronic disease, as this differs from, e.g., acute and life-threatening conditions or euthanasia of healthy animals.

Participants were not asked to explicitly differentiate between cat welfare, wellbeing, and quality of life, concepts that were central to reasoning, perceptions, and decisions, and used them somewhat interchangeably (as reflected in the analysis). The authors recognise that different orientations regarding animal welfare exist, including definitions or quantifications of quality of life [61,93]. The authors acknowledge that potentially variable and individual interpretations of these constructs introduce a layer of interpretive complexity to the narratives. The relationship between the research questions and welfare-related concepts was neither prospectively defined nor further explored. Future research would benefit from directly investigating how small animal veterinarians conceptualise animal welfare and quality of life in the context of diabetes mellitus in cats, as these definitions likely shape reasoning and interpretations of professional responsibility.

## Supporting information

S1 Appendix Additional information on methodological and analytical considerations. (DOCX)

S2 Appendix Interview guide. (DOCX)
