# Supplementary material for: To treat or not to treat: Experiences and considerations of veterinarians in management of cats with diabetes mellitus
Source: PLoS One. 2026 Feb 5;21(2):e0341762. doi: 10.1371/journal.pone.0341762 (PMC12875475; doi:10.1371/journal.pone.0341762)
Supplement: S1 Appendix — (DOCX) [file pone.0341762.s001.docx]

**S1 Appendix. Additional information on methodological and analytical considerations.**

In this appendix, supplementary information on methodological considerations and procedures is provided.

**Methodological framework**

Professional decision-making in veterinary practice includes considerations for both the animal and the owner. Beyond clinical factors such as prognosis and available medical options, how the veterinarian perceives and act in a specific situation is influenced by the perception of this situation and prior experiences and personal viewpoints (e.g., on our responsibilities towards animals). To gain a deeper understanding of associated issues, including what drives human behaviour, it is essential to consider individuals’ subjective perspectives (1). Qualitative research approaches is suitable for examining these nuanced dimensions (2). The research questions in this study were generated within a constructivist theoretical framework, aiming at presenting the lived experiences and rich meanings of participating veterinarians. We wanted to emphasize the individuality and diversity of participants’ experiences, giving them a key role in forming the overall narrative. Therefore, an experiential orientation towards the data was applied. In practice, this meant that the researcher engages deeply with the specific context to offer rich and situationally grounded interpretations of the topic.

The research team reasoned that the study would benefit from a reflexive approach (3, 4). Thus, the authors’ own experience and familiarity with the topic contributed valuable subjectivity to the analytic process. This informed both data collection and analysis, helping to ensure that participants’ own perspectives and subjectivities were reflected in the issues discussed during the interviews (3).

**Interview guide and interviews**

Individual in-depth semi-structured interviews were chosen for data collection, as it enables both capturing depth and complexity of lives experiences, as well as the opportunity for flexibility in questions and follow-up enquiries (4, 5). The interview guide was piloted with two veterinarians, both female, who had clinical experience cats diagnosed with diabetes mellitus (DM) from both smaller veterinary practice and referral clinics. The pilot interviews were conducted using the digital video conferencing platform Zoom and were reviewed by the first author and a co-author to assess question clarity and comprehension, as well as whether the questions were formulated to encourage participant engagement in a way that related to the research questions. The interview guide was adjusted based on these evaluations. In addition, the guide was further revised in response to unforeseen answers to questions about ethical challenges, e.g., where participants raised the topic of coping strategies which led to the inclusion of a follow-up question: “How do you cope or manage the challenges?”. Although all questions in the guide were covered during the interviews, their sequence varied depending on the flow of topics and lines of argumentation. Additional probing questions were asked when appropriate.

The interviews were conducted in Swedish via Zoom. Verbal consent for video recording was obtained from all participants and documented in writing by the interviewer (the first author). As video recording began only after consent had been given, the consent itself was not recorded. Participants were informed via Zoom when the recording started, notified about the recording icon and its meaning, and this was documented on video. They had also received prior information regarding how the interviews were to be executed, allowing time to consider their participation. One participant preferred not to be video recorded; therefore, only audio was captured in that case. Significant attention was given to establish good rapport (6). This included starting the interview with a broader question of more general character and avoiding phrasings that could be interpreted as leading, as well as ensuring a supportive interview atmosphere and by providing clear information about the interviewer (the first author), the research project and its purpose.

The number of participants was assessed stepwise and continuously during the course of data collection and was guided by the concept of information power (7), where the richness and the relevance of information in the dataset apprises the number of participants, and Braun and Clarke’s reflections on data collection within reflexive thematic analysis (8). The exploratory approach and the wider research questions, the broad inclusion criteria, and the analytic methodology (reflexive thematic analysis) indicated the need to include a larger number of participants. In contrast, the richer data items (in-depth individual interviews) and the research group´s diverse experience of the subject, generally entail fewer participants. First, five interviews were conducted with participants recruited from one of the two Facebook groups, and field noted were used to assess the data for differing experiences and data quality in relation to the research questions. Based on this and in search to diversify perspectives, two veterinarians were directly approached by the primary author in the Facebook groups, and, later, two additional veterinarians were recruited through personal contacts. After nine interviews, the data was assessed as rich enough to proceed with analysis. Discussions within the research group facilitated this assessment. However, to avoid prematurely closing data collection, one additional interview was conducted. Transcription and coding were postponed until all interviews were completed.

The recorded video interviews were transcribed manually and verbatim by the first author, using the software oTranscribe. To enhance transcript quality, structured descriptions for non-verbal occurrences (e.g. laugh, nod) was applied (9). Relevant excerpts and quotations from the transcripts were later translated into English by the first author. Pseudonyms were assigned to all participants to ensure confidentiality during analysis and reporting.

**Analytical process**

A six-phase indictive reflexive thematic analysis was used, guided by the steps described by Braun and Clarke (3, 10). Initially, NRZ (the first author) read through the transcripts to become familiar with each participant’s experiences, annotating preliminary observations and potential areas of analytical interest. The transcripts were then examined systematically and iteratively, with data excerpts that might be relevant to addressing the research questions coded in an inclusive manner. During this initial stages of coding, code refinement, and code organization, the software Delve was used. Surface-level coding was applied to capture what participants explicitly expressed, while an interpretive approach was adopted where deeper understanding was needed. As the analysis progressed, the coding evolved from a primarily semantic focus toward also including latent meanings. In total, the dataset was coded in three rounds. Two transcripts were independently coded by both the first author and a co-author, who is not a veterinarian. This co-author brought additional experiences, knowledge, and perspectives to the coding process, providing an alternative lens that enriched interpretation. These transcripts were selected because they represented differing veterinary perspectives and were considered likely to benefit from fostering additional reflexivity and critical engagement. The co-coded transcripts were read side-by-side by the main author, and variations in coding and interpretations were discussed within the research group. This process subsequently informed the coding of the remaining transcripts. The generated codes in the dataset were iteratively refined, renamed and elaborated, and new codes were developed and added where relevant. Related codes were collated and relevant data extracts organised together.

Once the initial phases of coding was complete, the codes were clustered into broader patterns of shared concepts and meaning (candidate themes). These were, again, examined in relation to the research questions and how they might alleviate our understanding of the issue. For this step, printed copies of the codes were utilized to provide an alternative visualization that supported the development of themes. Collaborative phases of the data analysis were supported by sessions using a whiteboard to create thematic maps, a process that facilitated the visualization of patterns and enhanced discussions within the research group. By going through the associated codes and data excerpts, the viability of the candidate themes were evaluated. Special attention were given to ensuring that candidate themes represented shared meanings across the dataset and participants and not constituting ideas of a single code. Through this process, three preliminary themes were generated, conceptualizing broader meanings of how veterinarians’ relate to the owner and the cat, and how they approach adaptations in disease management and protecting the cat from suffering. During discussions in the research group, it became clear that veterinarians’ perceived responsibilities towards the cat and the owner demonstrated a broader concept of sense-making that anchored the other themes together. After reviewing the candidate themes, the next step involved defining and naming the themes and engaging in a deeper analysis of each theme, forming an analytic story. At this stage, engagement with existing research situated the data and deepened the analysis. Throughout the analytical process, the diverse experiences and perspectives within the research group helped to enhance and broaden both the analysis and the resulting insights. See Figure 1 for an overview of the analytic process.


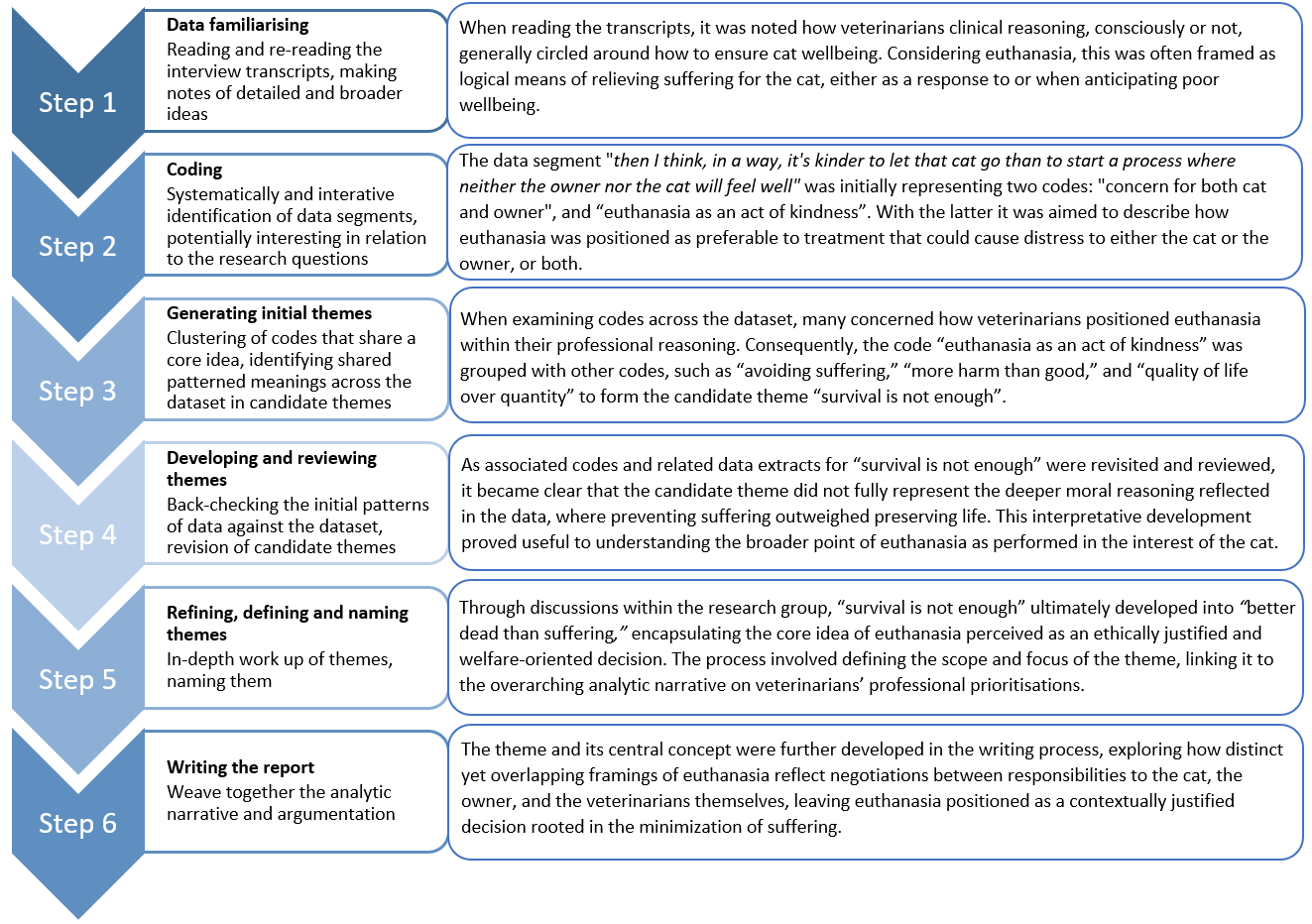


Figure 1. Overview of the thematic analytic process, including examples of theme development. Analytic steps are adapted from Braun and Clarke (11).

**Reflexivity**

In reflexive thematic analysis, the researcher is centered as an integral part of the research process, with their interpretations, choices, and positioning inevitably shaping the analysis and rendering it inherently subjective (3). Accordingly, discussions within the research team aimed to foster reflexivity, deepen interpretations and broaden perspectives, rather than to achieve coding consensus or pursue a single “accurate” representation of the phenomenon. The reflexive process, which included reflections on researcher positionality and how it may influence data engagement (3), was primarily informed by the first author (NRZ), a female PhD student with training in conducting qualitative research interviews and qualitative analysis of data. NRZ’s clinical experience as a small animal veterinarian with experience with DM in cats informed the analytical process. Familiarity with clinical management and the complexities of DM served as a resource (12), helping to resonate with participants’ accounts. While recognising researcher subjectivity, care was given to keep the coding process and theme development not influenced by pre-existing ideas and definitions from previous research, facilitating a data-driven and inductive approach. In this process, ongoing reflection and regular discussions within the research group was undertaken throughout the analytical process. This included considering what aspects of the data elicited particular interest, and how personal context and clinical experiences influenced that response, aiming to remain open to the veterinarians’ experiences and to explore their reasoning without preconceived assumptions. As to ensure inclusivity and to avoid overemphasizing areas of personal relevance, sparsely coded sections were revisited during analysis.

**References**

1. Höglund Nielsen B, Granskär M. Tillämpad kvalitativ forskning inom hälso- och sjukvård: Studentlitteratur; 2017.

2. Pope C, Mays N. Qualitative Research: Reaching the parts other methods cannot reach: an introduction to qualitative methods in health and health services research. BMJ. 1995;311(6996):42–5.

3. Braun V, Clarke V. Reflecting on reflexive thematic analysis. Qualitative Research in Sport, Exercise and Health. 2019;11(4):589–97.

4. Braun V, Clarke V. Conceptual and design thinking for thematic analysis. Qualitative psychology. 2022;9(1):3.

5. Kvale S, Brinkmann, S. Interviews: Learning the craft of qualitative research interviewing: SAGE Publications Inc.; 2009.

6. Dicicco-Bloom B, Crabtree BF. The qualitative research interview. Medical Education. 2006;40(4):314–21.

7. Malterud K SV, Guassora AD. Sample Size in Qualitative Interview Studies: Guided by Information Power. Qualitative Health Research. 2016;26(13):1753–60.

8. Braun V, Clarke V. To saturate or not to saturate? Questioning data saturation as a useful concept for thematic analysis and sample-size rationales. Qualitative Research in Sport, Exercise and Health. 2021;13(2):201–16.

9. Poland BD. Transcription quality as an aspect of rigor in qualitative research. Qualitative inquiry. 1995;1(3):290–310.

10. Braun V, Clarke V. Using thematic analysis in psychology. Qualitative Research in Psychology. 2006;3(2):77–101.

11. Braun V, Clarke V. Thematic analysis: A practical guide. Sage London; 2022.

12. Gough B, Madill A. Subjectivity in psychological science: from problem to prospect. Psychological methods. 2012;17(3):374.
