# Supplementary material for: To treat or not to treat: Experiences and considerations of veterinarians in management of cats with diabetes mellitus
Source: PLoS One. 2026 Feb 5;21(2):e0341762. doi: 10.1371/journal.pone.0341762 (PMC12875475; doi:10.1371/journal.pone.0341762)
Supplement: S2 Appendix — (DOCX) [file pone.0341762.s002.docx]

**S1 Appendix. Semi-structured interview guide.**

## How often do you see cats with diabetes mellitus? What are your thoughts on them as patients?

## How do owners usually react when their cat is diagnosed with diabetes?

## Why do you think they react that way?

## How do you usually respond?

## What factors do you consider when deciding on a treatment plan?

## Do you and the owner usually agree on the treatment plan?

## Have there been occasions when you needed to compromise on your treatment plan?

## How would you describe good quality of life in a cat, generally speaking?

## How important is the cat’s quality of life when recommending treatment or euthanasia?

## Have you experienced cases where the cat was euthanised at the time of diagnosis?

## If yes: Would you like to share more about that?

## Did you and the owner agree on the decision?

## Have there been times when you and the owner did not agree on the decision to euthanise, at other stages of diabetes management?

## Why do you think you didn’t agree?

## When do you think euthanasia is the right decision?

## Do you find it a difficult decision to make?

## When do you think euthanasia is not the right decision?

## How do you view the challenges associated with managing diabetic cats?

## How do these challenges affect you?

## How do you cope with or manage them?

## As a vet, one might argue that responsibility lies with both the cat and the owner. How do you view this balance?

## How do you think your workplace influences the decisions you make? Are there any established norms or traditions?

## What do you do when faced with a diabetes case that you find ethically challenging?

*Please note that, due to the semi-structured nature of the interviews, additional questions, probing, and follow-ups were incorporated as needed. Furthermore, the order of the questions in the guide is intended to be only a guideline and was adjusted to follow the natural flow of each interview.*
